# Schwartz rounds in undergraduate medical education facilitates active reflection and individual identification of learning need

**DOI:** 10.15694/mep.2018.0000230.1

**Published:** 2018-10-15

**Authors:** Claire Stocker, Andrew Cooney, Peter Thomas, Bharathy Kumaravel, Kenneth Langlands, Jasmine Hearn

**Affiliations:** 1 University of Buckingham

**Keywords:** Schwartz rounds, reflection, undergraduate medical education

## Abstract

This article was migrated. The article was marked as recommended.

Strategies applying Schwartz Rounds to improve wellbeing of medical students has focused on the clinical years of study. This pilot study investigates whether Schwartz Rounds could be effective in developing students’ reflective practice in Year 2 undergraduates. Engagement with the Schwartz Round was high with over 50% of the students identifying learning needs through reflection on the Round. Schwartz Rounds promoted recognition of the value of reflective practice and increased self-awareness of student needs.

## Introduction

Changing healthcare provision in the UK has altered the demography of medical students ( Appleby *et al*, 2011). Growing admissions and reduced funding have stretched the healthcare system creating pressured work and education environments. Resulting stress can negatively affect welfare by increasing risk of depression ( Schwenk *et al*, 2010) and burnout ( Almeida *et al,* 2016). UK Medical School attrition has been calculated as high as 14% ( O’Neill *et al*, 2011) and whilst drop-out rates are multifactorial, mental health is a significant factor. Medical students are at particular risk of developing mental health problems while learning and must be given better support to prepare them for emotional toil (BMA, 2018).

Kenneth Schwartz, a former US lawyer who died from lung cancer in his early 40s, wrote a passionate account about the positive impact of receiving compassionate care, but highlighted the emotional cost to the staff. Schwartz proposed creating safe spaces to reflect and share the psychological aspects of caring, dubbed Schwartz Rounds. The underlying premise is that to provide compassionate care, staff must feel supported in their work. Social circumstances compounded their innate attributes resulting in increasing adoption in the UK (Robert *et al*, 2015) and have been valued due to increasing respectful teamwork whilst reducing stress, thus reducing isolation and providing support (Chadwick *et al*, 2016).

Medical education should prepare our next generation of doctors for the reality and uncertainties of clinical practice ( Schön, 1983). To accomplish this; medical schools should be aware of the difficulties that students face, particularly the pressures and constraints from learning experiences outside the formal taught curriculum (the ‘hidden curriculum’). Introducing clinical medical students to Schwartz Rounds enhances empathy, resilience, team-working and reflection skills ( Barker *et al,* 2016; Gishen *et al,* 2016). Schwartz Rounds have been demonstrated to enhance communication skills in preclinical medical students ( Shield *et al*, 2011), but have not yet been evaluated for effectiveness in enhancing the other reported attributes in the early years of medical education.

This preliminary study utilizes the integrated nature and group work-led structure of the curriculum at the University of Buckingham’s Medical School to examine the proposal that the application of Schwartz Rounds in the early years of undergraduate medical education might be a means of developing the student’sawareness of their reflective practice, and whether a more detailed investigation into the benefits of Schwartz Rounds in pre-clinical medical education is warranted.

## Methods

Medical educators introduced the topics with a personal experience and then facilitated the Schwartz Round. 83 second year students in teams of 6 - 8 discussed their topic for 10-15 minutes before presenting. All participants were then invited to share their thoughts, questions and experiences.

Topic 1: Change and resilience: think about the difficulty in coming to a new healthcare environment and how you adapted.How did you feel introducing yourselves to patients, examining patients, considering your and their vulnerability?

Facilitators prompted discussions on 1.) Learning personal skills required for physical examinations 2.) Reflection on performance and skills 3.) Challenges in supporting students.

Topic 2: Duty of Candour: think about any adverse incidences, clinical or non-clinical, you have seen, non-clinical or clinical.Consider the safety implications to patients and colleagues.

Facilitators prompted discussions on identifying and overcoming challenges in clinical and medical school environments.

The students were given a feedback questionnaire to supply in white spaces how they thought the Round had impacted on specific aspects of their professional identity. The questions and feedback analysis are shown in table 1. Statistics were analysed by Pearson’s Chi Squared test using SPSS.

## Results/Analysis

Feedback completion ranged from 68% to 97% for the individual questions ( table 1). Feedback suggested the Round positively impacted on each outcome for at least 90% of learners. There was no statistical correlation between completion percentage and positive response (p=0.41), indicating that the incomplete feedback did not bias the data. Between 26% and 50% of the responses for each category stated that a learning need had been identified. Positive responses were likely inversely related to identification of learning needs (88% chance, p=0.12) and the highest scoring category in positivity scored lowest in identifying learning needs, and vice versa.

**Table 1.** Feedback analysis.

Question	Completion (%)	Positivity of response (%)	Learning need identified (%)
Confidence in handling sensitive issues	97	90	50
Beliefs in the importance of empathy	84	92	38
Actual empathy with patients as people	68	90	33
Confidence in handling non-clinical aspects of care	74	96	26
Openness to expressing thoughts, questions, feelings	90	93	32

Student 1: *“interactive and useful session: great to share clinical experiences in a safe space and be able to listen to other experiences and feelings”.*

This quote echoed the responses of many students, who indicated that the non-judgemental safe space that was created improved confidence to share and acknowledgeeach other’s experiences and feelingsregarding managing sensitive clinical issues.

Student 2: *“awesome to know my classmates better through shared experiences and would love to participate in further rounds. It felt like we were fellow humans with stories rather than students”.*

Students benefitted from sharing stories with colleagues, which helped normalise and contextualise experiencesoutside the ‘medical student’ persona. This developed empathy leading to openexpressionof thoughts and feelings and reflective insight into the students’ own learning needs.

Student 3: *“The session is well-structured. Facilitator anecdotes helping students understand and explore resolutions to their own and others’ problems. It felt safe to discuss things personal to me. I’d happily attend another Schwartz Round”.*

Peer observations and staff experiencesenabledappreciation of the benefits of such sharing. Participating in the sharing built empathy, confidence and reflective insight into their own learning. Many students reflected on their willingness to participate in Schwartz rounds in future, indicating recognition of their need for outlets such as this, and the value of reflective practice.

Student 4: *“Not gained confidence as still lacking first-hand experience”.*

Negative responses tended to arise from a feeling of separation from the topics discussed, reducing engagement.

## Discussion

As with the earlier study in year 5 and 6 students ( Gishen et al, 2016), the Round was very well received. Percentage feedback was higher in this study: between 68% and 97% (year 2 students) compared to between 37% and 77% for year 5/6 students. The percentage of students regarding the rounds had a positive effect on their professional identity was also higher in the year 2 students (over 90% compared to over 80%) suggesting that teaching opportunities of this nature could be incorporated into the taught undergraduate curriculum and are acceptable to students at this early stage in their training.

At least 50% of learners’ reflection identified learning needs because of the Schwartz Round. Whilst the overwhelming response for all categories of reflection was positive it is curious that there was an apparent inverse relation between the extent of this positivity and the identification of learning needs. By raising awareness of learning needs the learners may feel that the Schwartz Round has not increased their capabilities. However, the realisation that Rounds can help students identify how they can develop is a positive step and the fact that this occurred in at least 50% of the learners is a positive reflection on the value and success of the Schwartz Round experiment.

A proportion of learners felt that their clinical inexperience reduced the effectiveness of the Rounds for them. Rounds are equally suited to non-clinical scenarios so future study should consider initially encompassing difficulties arising from the School environment and be mindful of the enhanced communication skills the students are expected to achieve ( Shield *et al*, 2011) when evaluating communication-mediated results.

## Conclusion

This pilot study showed that Schwartz Rounds early in undergraduate programs promote appreciation of reflective practice and increased self-awareness of requirements needed to support the student’s continuing professional development (CPD) and an extensive investigation into the benefits and implication methods is warranted.

## Take Home Messages

• Schwartz Rounds early in undergraduate programs promote appreciation of reflective practice
• Schwartz Rounds are well-recieved by pre-clinical medical students
• Schwatrz Rounds in early undergraduate programs may need to encompass non-clinical scenarios in addition to clinical scenarios to enhance effectiveness
• Further study on Schwartz Rounds in early undergraduate programs is warranted to investigate their other reported benefits in full

## Notes On Contributors

Dr Stocker: Senior Lecturer and Student Support Lead.

Dr Cooney: Clinical Director and Professionalism Theme Lead.

Dr Kumaravel: Senior Clinical Lecturer and Public Health Theme Lead.

Professor Thomas: AE Consultant, Senior Clinical Lecturer and Phase II Lead.

Dr Langlands: Senior Lecturer and Phase I Lead.

Dr Hearn: Lecturer and Health Psychology Lead.
