# Supplementary material for: Schwartz rounds in undergraduate medical education facilitates active reflection and individual identification of learning need
Source: MedEdPublish (2016). 2018 Oct 15;7:230. [Version 1] doi: 10.15694/mep.2018.0000230.1 (PMC10712006; doi:10.15694/mep.2018.0000230.1)
Supplement: Peer review history

# Reviewer report: Reviewer response for version 1

**DOI:** 10.21956/mep.19699.r29457

**Published:** 2018-10-22

**Authors:** John Cookson

**Affiliations:** 1 University of Worcester

This review has been migrated. The reviewer awarded 4 stars out of 5 I've heard of the principle but not the name. It seems to me that the students were reflecting on a theoretical scenario as I was not sure of the relation of the tutor's experiences to the questions. It should work even better when the students can reflect on their own clinical experiences. It is possible by working with a SP to recreate the scenario and try out different approaches.

Reviewer Expertise:

NA

No decision status is available

# Reviewer report: Reviewer response for version 1

**DOI:** 10.21956/mep.19699.r29456

**Published:** 2018-10-19

**Authors:** P Ravi Shankar

**Affiliations:** 1 American International Medical University

This review has been migrated. The reviewer awarded 4 stars out of 5 Before reading this paper I was not aware about Schwartz rounds but I have used many of the principles mentioned in this paper among students including beginning the sessions by having the facilitator share their personal experiences and creating a safe space where students could share their ideas and opinions. I am interested in knowing if there are specific characteristics associated with the Schwartz rounds and how these rounds differ from other debriefing and student engagement sessions. Were all the second-year participants in the same room? Did they present their findings to the entire group? The authors mention two topics which were used. Were these topics introduced sequentially? The manuscript is well-written but there are problems with spacing between words. The article would be of interest to medical educators.

Reviewer Expertise:

NA

No decision status is available
